# Variation in soil bacterial community characteristics inside and outside the West Ordos National Nature Reserve, northern China

**DOI:** 10.3389/fmicb.2024.1404848

**Published:** 2024-06-11

**Authors:** Pu Guo, Shuai Li, Jinlei Zhu, Qi Lu

**Affiliations:** ^1^Institute of Ecological Conservation and Restoration, Chinese Academy of Forestry, Beijing, China; ^2^Institute of Desertification Studies, Chinese Academy of Forestry, Beijing, China; ^3^Experimental Center of Desert Forestry, Chinese Academy of Forestry, Dengkou, China

**Keywords:** desert grassland, nature reserve, soil bacteria, plant biomass, soil chemical index

## Abstract

Nature reserves are crucial for protecting biological habitats and maintaining biodiversity. Soil bacterial community plays an irreplaceable role in the structure and function of ecosystem. However, the impact of nature reserves on soil bacterial communities is still unclear. To explore the effects of desert grassland nature reserve management on soil microbial communities, we compared the differences in soil bacterial community composition, α-diversity and community structure inside and outside a desert grassland nature reserve, and explored the correlation between soil bacterial communities and plant biomass and soil chemical index. We found that (1) the relative abundance of Acidobacteriota is highest in the soil both inside and outside the nature reserve in shrub grassland; (2) the Chao1 index of soil bacterial communities in the core protected zone and general control zone of the reserve was significantly higher than that outside the reserve (*p* < 0.05) in the shrub grassland. Similarly, in the herbaceous grassland, the Shannon index of soil bacterial communities was significantly higher in the core protected zone of the reserve than that outside the reserve (*p* < 0.05). (3) While we found no significant difference in soil bacterial community structure between inside and outside the reserve in the shrub grassland, we found that the soil bacterial community structure in the core protected zone was significantly different from that outside the reserve in the herbaceous grassland (*p* < 0.05); (4) we also found that higher plant productivity and soil nutrients promoted most soil dominant bacterial phyla, while higher soil pH and salinity inhibited most soil dominant bacterial phyla. Our findings thus help better understand the influencing factors of and the mechanisms behind variation in soil bacterial communities inside and outside desert grassland nature reserves.

## 1 Introduction

Desert grassland is the driest type of grassland vegetation. The desert grassland in Inner Mongolia Autonomous Region serves as a crucial base for animal husbandry and an important ecological security barrier in northern China (Guo et al., 2022). A nature reserve that focuses on protecting the natural ecosystem of desert biotic and abiotic environments is known as a desert ecosystem type nature reserve (Ma et al., 2023). These reserves are situated in fragile environments that suffer from issues such as overgrazing, drought, and land degradation (Sankey et al., 2015; Xiong et al., 2016; Zuo et al., 2022). Therefore, it is crucial to enhance the management and protection of these reserves. The West Ordos National Nature Reserve is home to a variety of rare plants, such as *Tetraena mongolica*, *Helianthemum songaricum*, *Potaninia mongolica*, *Reaumuria songarica*, which hold significant ecological value (Li E. G. et al., 2018; Dong et al., 2023).

Soil microorganisms play a crucial role in soil ecosystems (Griffiths et al., 2008; Xia et al., 2020). They reflect the health of soil ecosystems (Agbenin and Adeniyi, 2014) and are essential for soil material cycling, energy flow, and promoting plant growth and development (Mlambo et al., 2007; Zhang et al., 2023). Soil bacteria are a crucial component of soil microorganisms. Numerous studies have been conducted on the characteristics of soil bacterial communities, with a primary focus on the impact of natural and anthropogenic factors on their structure and function. These factors include climate change, plant community succession, changes in land use types, alterations in the physical and chemical properties of the soil (Li G. et al., 2018; Ma S. et al., 2018; Chai et al., 2019; Sünnemann et al., 2021). For instance, in alpine meadows, soil bacterial biomass typically first decreases and then increases with grazing intensity (Yang et al., 2018). In semi-arid meadows, heavy grazing may significantly reduce soil bacterial biomass (Liu et al., 2016). These findings suggest that the impact of human activities on soil bacterial communities could vary with vegetation type. Studying the role and mechanism of nature reserves in protecting the structure and function of soil bacteria can guide the improvement and development of conservation measures. Some studies have focused on the impact of nature reserves on plants and animals, while others have studied changes in land use and ecosystem services within and outside nature reserves, and some studies have found that nature reserves can enhance local plant diversity and ecosystem services (Tranquilli et al., 2012; Carranza et al., 2013; Gray et al., 2016; Yu et al., 2024). However, few studies have compared the variation in characteristics of soil bacterial communities inside and outside nature reserves. Even a comparative study of soil bacterial communities in core protected areas and general control areas in nature reserves has not been reported. Based on previous studies, nature reserves enhance local plant diversity and ecosystem services, which may have a positive impact on soil bacterial communities. However, the impact and mechanism of management measures in nature reserves on soil microorganisms are still unclear. Therefore, studying the structure and function of soil bacterial communities inside and outside nature reserves is significant for the sustainable management and optimization of soil ecosystems (Atwell et al., 2018).

In this study, we systematically and comprehensively compare the differences in soil bacterial community characteristics between inside and outside the West Ordos National Nature Reserve using ground survey data. We also explore the relationship between soil bacteria and soil physicochemical properties and plant productivity. Our aim is to provide a reference basis for scientifically and objectively evaluating the effectiveness of soil ecosystem protection in desert nature reserves in arid zones and upgrading the management level of the nature reserves.

## 2 Materials and methods

### 2.1 Study site

**Figure 1:**
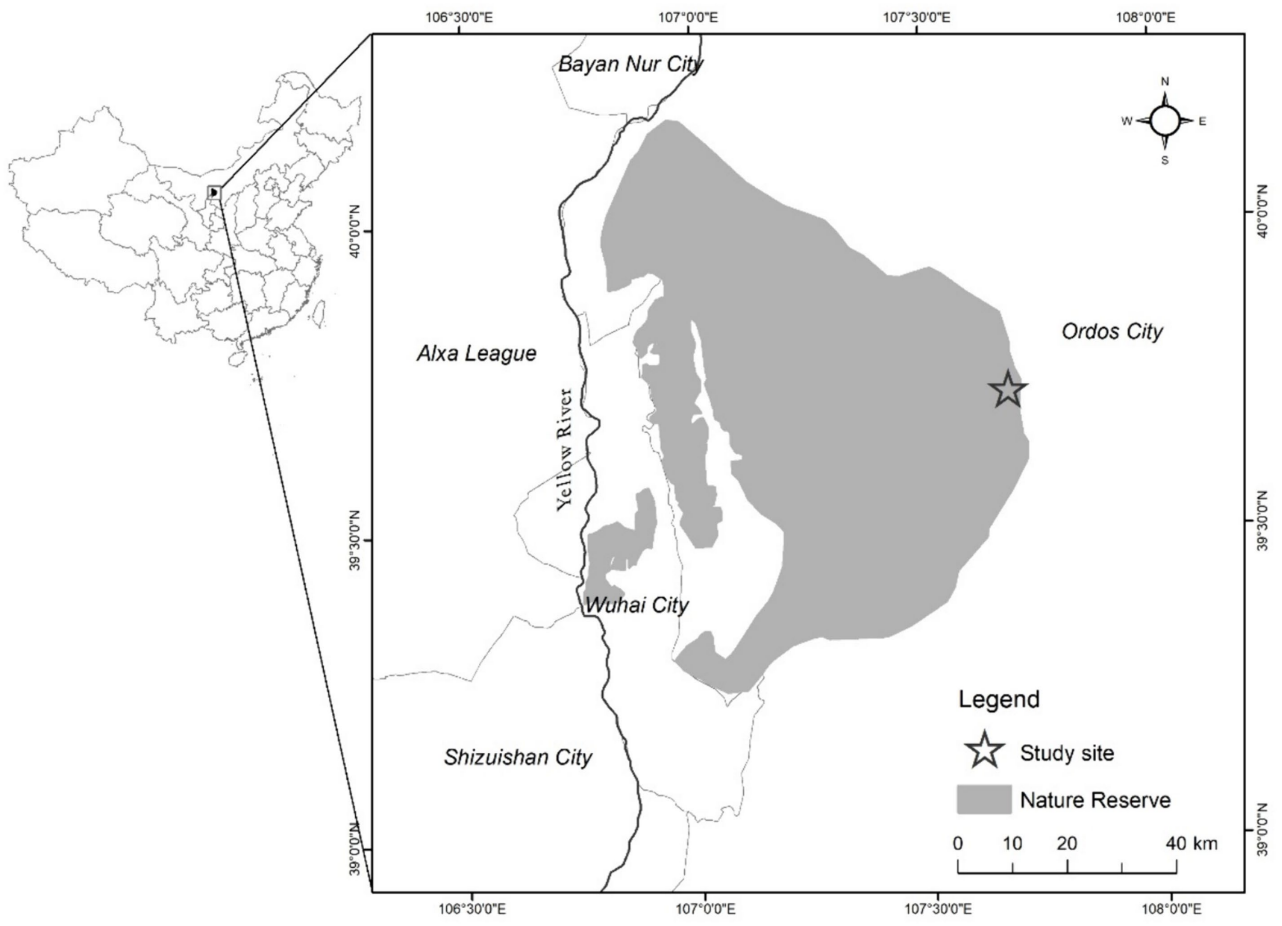
Location of the study area.

The research area is situated in the West Ordos National Nature Reserve (106°40′–107°44′ E, 39°14′–40°11′ N, 1,100–2,000 m asl), Otog Banner, Inner Mongolia Autonomous Region (Figure 1). The area has an arid and semi-arid continental climate in the mesotemperate zone. The winter is long and cold, the summer is short and warm, the spring is windy, and the fall has stable weather. The frost-free period in this area is 129 days. The average annual temperature is 7°C, with the lowest temperature recorded at −36.8°C and the highest at 37°C. Annual precipitation averages at 272.3 mm, with uneven distribution throughout the year, mostly concentrated in June–September. Annual evaporation is 2470.4 mm, which is 9.1 times the amount of precipitation. The average annual wind speed is 3 m/s. The average wind speed in the area is 3.4 m/s, with an average of 36 days of winds of grade 6 or above in a calendar year. Of these days, 19, or 52.5%, occur in spring. The soil is mainly chestnut-calcium soil. The main plant species in the area include *Zygophyllum xanthoxylum*, *Tetraena mongolica*, *Helianthemum songaricum*, *Reaumuria songarica*, and *Stipa tianschanica*. Established in 1995, West Ordos National Nature Reserve primarily protects endangered plants and desert ecosystems, such as *T. mongolica* and *H. songaricum*. It is classified as a desert ecosystem nature reserve.

### 2.2 Sample selection and setting

Nature reserves are divided into core protected zones and general controlled zones. The core protected zones are primarily responsible for limiting human activities to the greatest extent possible in order to fulfill their protection function. The general controlled zones, on the other hand, are responsible for fulfilling their protection function while also considering public service functions such as scientific research, education, and recreation. In this research area, the core protected zone is closed and human activities are strictly prohibited. Necessary human activities, such as grazing, may be limited to the general control zone. Production activities outside the reserve are not restricted. The primary human activities in this study area are cattle and sheep grazing and crop planting. The plant biomass and diversity of the core protected area in the nature reserve are higher and more abundant, followed by the general control area, while the plant biomass and diversity outside the nature reserve are lower than those inside the protected area. The degree of soil desertification outside the protected area is more serious than that inside the protected area, and there is obvious soil salinization. The survey was conducted in August 2021, with sample plots selected from the core protected zone, general controlled zone, and outside the reserve. And the three areas are 30 km apart from each other. Two vegetation types, shrub grassland and herbaceous grassland, were surveyed in each sample site. Soil surveys were conducted in three randomly selected squares of each planting type, separated by 500 meters from each other, for a total of 18 squares. The “S”-shaped five-point mixed sampling method was used to collect soil samples from each plot at depths of 0 to 20 cm, and the samples were then mixed to create a homogeneous sample of the site (Wei et al., 2024). The soil samples were divided into two portions. One portion was used to determine soil physicochemical properties, while the other was stored in a refrigerator at −80°C after impurities were removed. This portion was used for high-throughput sequencing of soil bacteria (Teng et al., 2018).

### 2.3 Sample collection and determination method

The biomass survey for herbaceous plants involved cutting the aboveground portion of all grasses in the sampling plots by species. The cut grasses were then brought back to the laboratory, dried in an oven at 65°C to a constant weight, and weighed. For shrubs, standard plants of representative sizes and heights were selected from the surveyed sampling plots. The aboveground portion of the standard plant was cut near the ground using scissors. The samples were then placed in paper bags and taken to the laboratory for biomass dry weight determination using the drying method. Sample biomass was calculated using the formula: biomass of shrubs = biomass of standard plants × number of shrub plants. Following plant sampling, a single root sample measuring 0–40 cm was taken from each sample plot using a root drill. The root samples were rinsed with water using a 2 mm sieve. After rinsing, the root samples were dried in an oven at 65°C until a constant weight was achieved. The weight was then used to calculate the belowground biomass of the plants. Soil pH, salinity, organic carbon, total nitrogen, and effective phosphorus were measured using a pH meter, conductivity analysis, oxidation of potassium dichromate with external heating, automatic nitrogen fixation, and ICP-AES analysis, respectively (Crepin and Johnson, 1993).

### 2.4 Extraction, sequencing and bioinformatics analysis of soil bacterial DNA

In the laboratory, soil samples were subjected to DNA extraction and PCR amplification. The PowerSoil DNA extraction kit (MoBio Inc., United States) was used to extract total soil DNA from 0.3–0.5 g of soil samples, following the experimental steps specified in the kit. The gene fragments of the V3–V4 region were amplified using primers 338F (5′-ACTCCTACGGGGAGGCAGCA-3′) and 806R (5′-GGACTACHVGGGTWTCTAAT-3′). The PCR reaction system consisted of 5 × FastPfu Buffer (4.0 μL), dNTPs (2.5 mmol/L) (2.0 μL), forward and reverse primers (5 μmol/L) (0.8 μL each), FastPfu Polymerase (2.5 U/μL) (0.4 μL), BSA (0.2 μL), Template DNA (10 ng), and ddH_2_O (supplemented to 20 μL). PCR reaction conditions were as follows: 95°C for 5 min; 25 cycles of 95°C for 30 s, 55°C for 30 s, and 72°C for 40 s; 72°C for 7 min, and 4°C for infinite conservation. The amplification products were loaded onto a 3% agarose gel and visualized using a gel imaging system. No changes in content were made. The PCR products were then quantified using the Qubit fluorescence quantification system and mixed in the appropriate proportion for sequencing. MiSeq library construction was performed followed by online sequencing using Illumina HiSeq2500 (Liu et al., 2014). The data for each sample was split based on the barcode sequence. The barcode and primer sequences were then spliced using FLASH (v1.2.7) pairs (Magoc and Salzberg, 2011). The high-throughput sequence data were processed using QIIME (v1.7.0). Valid sequences were clustered using UPARSE (v7.0) (Caporaso et al., 2013). The sequences were clustered to obtain operational taxonomic units (OTUs) using a default 97% sequence similarity. From each OTU, one representative sequence was selected, and the species annotation was performed using the RDP Classifier with the Greengenes database as a reference at a confidence level of 80% (Caporaso et al., 2010). Finally, the sample data were standardized to the lowest number in the sample.

### 2.5 Statistical analysis

We use relative abundance to characterize the dominance of soil bacterial phyla and orders. The higher the relative abundance of soil bacterial phyla and orders, the higher the degree of dominance. In this study, the top 10 bacterial phyla and order in relative abundance were selected as the dominant bacteria. The soil bacterial community’s α-diversity was measured using the Chao1 index and Shannon-Wiener index (Jones, 2010). The Chao1 index was calculated using the formula:

Chao1 index:


$$\mathrm{Chao}1=S_{obs}+\frac{F_{1}\left(F_{1}-1\right)}{2\left(F_{2}+1\right)}$$

“*S*_obs_” denotes the number of OTUs observed in the sample. “*F*_1_” and “*F*_2_” denote the number of OTUs when there is only one sequence and when there are two sequences, respectively.

The Shannon-Wiener index was calculated using the formula:

Shannon-Wiener index:


$$H=-\overset{S_{obs}}{\underset{i=1}{\sum }}\frac{n_{i}}{N}\ln\frac{n_{i}}{N}$$

“*S_obs_*” denotes the number of OTUs observed in the sample. “*n_i_*” is the number of OTUs containing “*i*” sequences. “*N*” is the number of OTUs for all sequences.

The Chao1 index is used to estimate the number of OTUs that are present in a sample. The Shannon-Wiener index is used to measure the diversity of a community. In the formula, n is the total number of bacteria of a particular species and *N* is the total number of bacteria of all species. The α-diversity was calculated using *R* and single variance analysis of soil bacterial alpha diversity index was performed in different regions, and multiple comparisons were made with Tukey HSD. Principal component analysis was used to analyze the differences in soil bacterial community structure between core protected areas, general control areas and outside protected areas, and permutational multivariate analysis of variance was used for statistical testing. Spearman’s method was used to perform correlation analysis, including positive and negative correlation, based on the abundance of each species and the changes. Statistical tests were then conducted to filter out data groups with absolute values of correlation coefficients greater than 0.1 and *p* ≤ 0. 05. In this study, correlation network analysis graphs were created using the Python language to demonstrate the top twenty phyla with the highest correlation. The analysis was performed in Cytoscape 2.6 (Otasek et al., 2019). We define the bacterial phylum with the largest number of relationships with other bacterial phyla as the core bacterial phyla. If some bacterial phyla have the same number of correlations with other bacterial phyla, the one with the highest relative abundance is selected as the core bacterial phyla. A correlation heatmap was created using Spearman’s correlation coefficient to analyze the correlation between the top 10 dominant bacterial phyla and orders in soil and both plant productivity and soil factors.

## 3 Results

### 3.1 Comparison of dominant bacterial phyla, orders and α-diversity of soil bacterial communities in and outside nature reserves

#### 3.1.1 Dominant phyla and orders in the soil bacterial community

**Figure 2:**
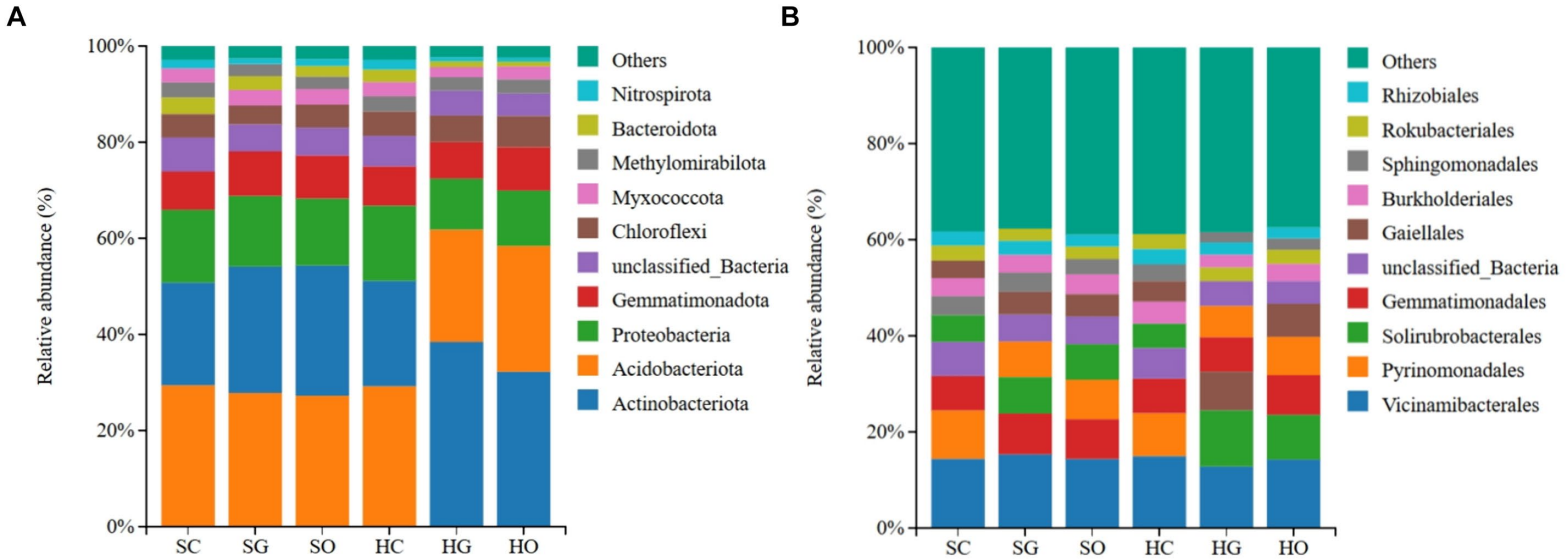
Distribution of soil dominant bacterial phyla **(A)** and orders **(B)** inside and outside protected areas. SC, shrub grassland in core protected zone; SG, shrub grassland in general controlled zone; SO, shrub grassland outside the protected area; HC, herbaceous grassland in core protected zone; HG, herbaceous grassland in general controlled zone; HO, herbaceous grassland outside the protected area.

In the shrub grassland, the top three dominant bacterial phyla of the soil in the core protected zone, general controlled zone, and outside the protected areas were Acidobacteriota (29.28 ± 2.03, 27.22% ± 1.22, 29.21% ± 2.42%), Actinobacteriota (21.47% ± 1.85, 26.36% ± 2.61, 27.11% ± 2.61%) and Proteobacteriota (15.23% ± 2.00, 14.80 ± 4.35, 14.07% ± 4.14%), respectively. In herbaceous grassland, the top 10 dominant bacterial phyla in the soil of the core protected zone were Acidobacteriota (29.21% ± 2.42%), Actinobacteriota (21.99% ± 1.93%), and Proteobacteria (15.61% ± 1.26%); and the dominant bacterial phyla in the soil of the general controlled zones and the soils outside the protected areas were Actinobacteriota (38.58% ± 3.84 and 32.36% ± 4.76%), Acidobacteriota (23.35% ± 1.45 and 26.06% ± 3.65%) and Proteobacteria (10.58% ± 2.09 and 11.48% ± 1.39%) in general control areas and outside protected areas, respectively (Figure 2A).

In the shrub grassland, the top three dominant bacterial orders in the soil of the core protected zone were Vicinamibacterales (14.33% ± 1.78%), Pyrinomonadales (10.02% ± 1.28%) and Gemmatimonadales (7.19% ± 0.34%); the top three dominant bacterial orders in the soil of the general controlled zone were Vicinamibacterales (15.34% ± 0.22%), Gemmatimonadales (8.47% ± 0.69%) and Solirubrobacterales (7.59% ± 1.12%); the top three dominant bacterial orders of soils outside the protected area were Vicinamibacterales (14.35% ± 0.98%), Gemmatimonadales (8.22% ± 1.09%) and Pyrinomonadales (8.22% ± 1.60%). In herbaceous grassland, the top three dominant bacterial orders in the soil of the core protected zone were Vicinamibacterales (14.96% ± 1.33%), Pyrinomonadales (8.97% ± 1.48%) and Gemmatimonadales (7.18% ± 0.35%); the top three dominant bacterial orders in the soil of the general controlled zone were Vicinamibacterales (12.84% ± 0.93%), Solirubrobacterales (11.71% ± 1.10%) and Gaiellales (8.07% ± 1.29%); and the top three dominant bacterial orders of soils outside the protected areas were Vicinamibacterales (14.18% ± 1.94%), Solirubrobacterales (9.34% ± 1.63%) and Gemmatimonadales (6.92% ± 1.38%) (Figure 2B).

#### 3.1.2 Alpha diversity of the soil bacterial community

**Figure 3:**
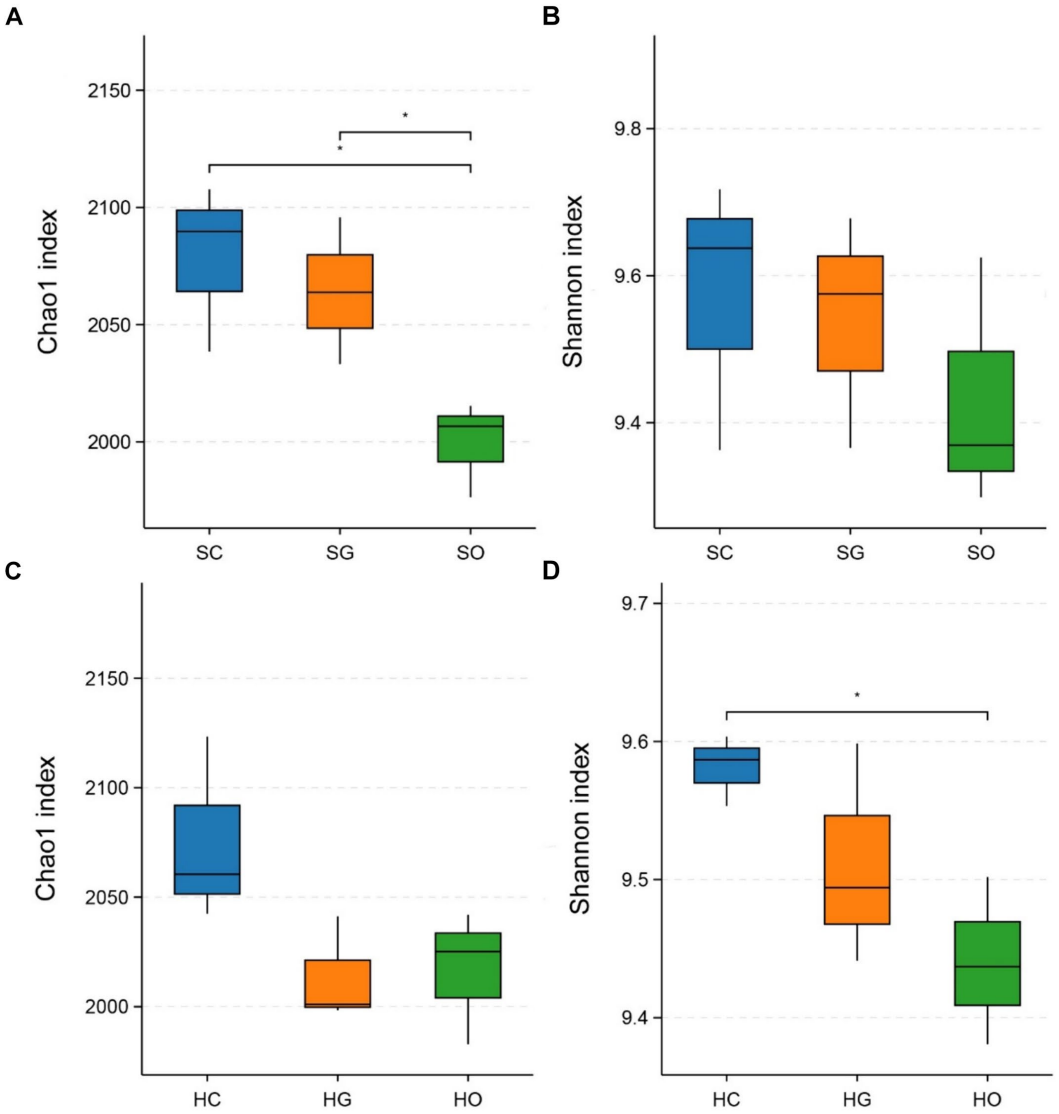
Comparison of chao1 index and Shannon index of shrub grassland **(A,B)** and herbaceous grassland **(C,D)** inside and outside the nature reserves. SC, shrub grassland in core protected zone; SG, shrub grassland in general controlled zone; SO, shrub grassland outside the protected area; HC, herbaceous grassland in core protected zone; HG, herbaceous grassland in general controlled zone; HO, herbaceous grassland outside the protected area.

Figure 3 displays the α-diversity indices of soil bacterial communities inside and outside the protected areas. In the shrub grassland, the Chao1 indices of soil bacterial communities in both core protected zones and general controlled zones were significantly higher than those outside the protected areas (*p* < 0.05) (Figure 3A). The soil bacterial communities’ Shannon index was higher in both core protected zones and general controlled zones than outside the protected areas (Figure 3B). In herbaceous grassland, the Chao1 index of soil bacterial communities was higher in core protected zones than in general controlled zones and outside protected zones (Figure 3C). The Shannon index indicated that the soil bacterial community in core protected zones had a significantly higher diversity than that in outside protected zones (*p* < 0.05), with general controlled zones falling in between (Figure 3D).

### 3.2 Comparison of soil bacterial community structure inside and outside the nature reserve

#### 3.2.1 Principal component analysis of soil bacterial community structure

**Figure 4:**
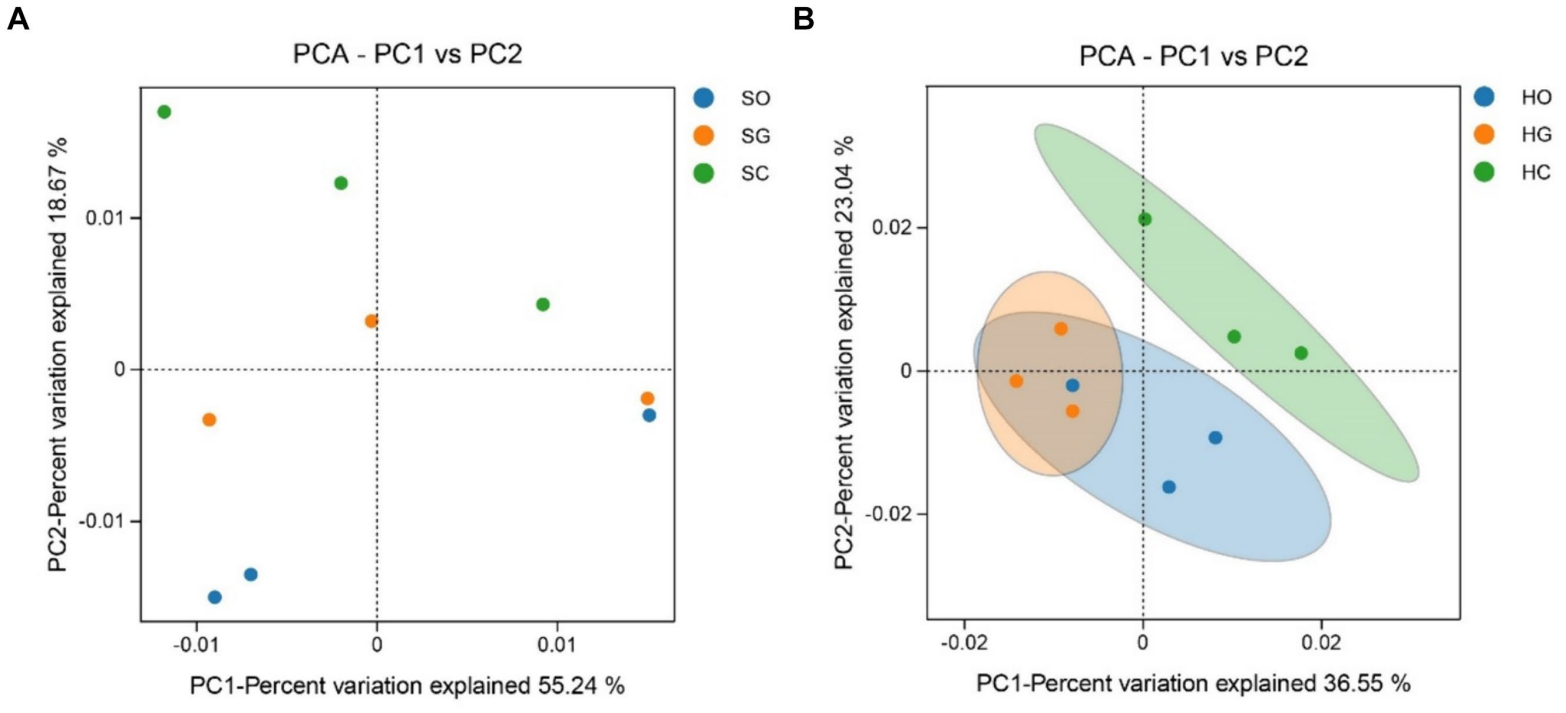
Analysis of the principal components of shrub grassland **(A)** and herbaceous grassland **(B)** inside and outside nature reserves. SC, shrub grassland in core protected zone; SG, shrub grassland in general controlled zone; SO, shrub grassland outside the protected area; HC, herbaceous grassland in core protected zone; HG, herbaceous grassland in general controlled zone; HO, herbaceous grassland outside the protected area.

The soil bacterial community structure in the shrub grassland was not significantly different inside and outside the protected area, although the sum of the contribution of the first and second principal components to the difference in soil bacterial community structure was 73.91% (Figure 4A). The first and second principal components contributed to 59.59% of the difference in soil bacterial community structure in the herbaceous grassland (Figure 4B). There was a significant difference (*p* < 0.05) between the soil bacterial community structure inside and outside the protected area, indicating that the establishment of the protected area had a significant impact on the soil bacterial community structure of the herbaceous grassland.

#### 3.2.2 Correlation network analysis of soil bacterial phyla

**Figure 5:**
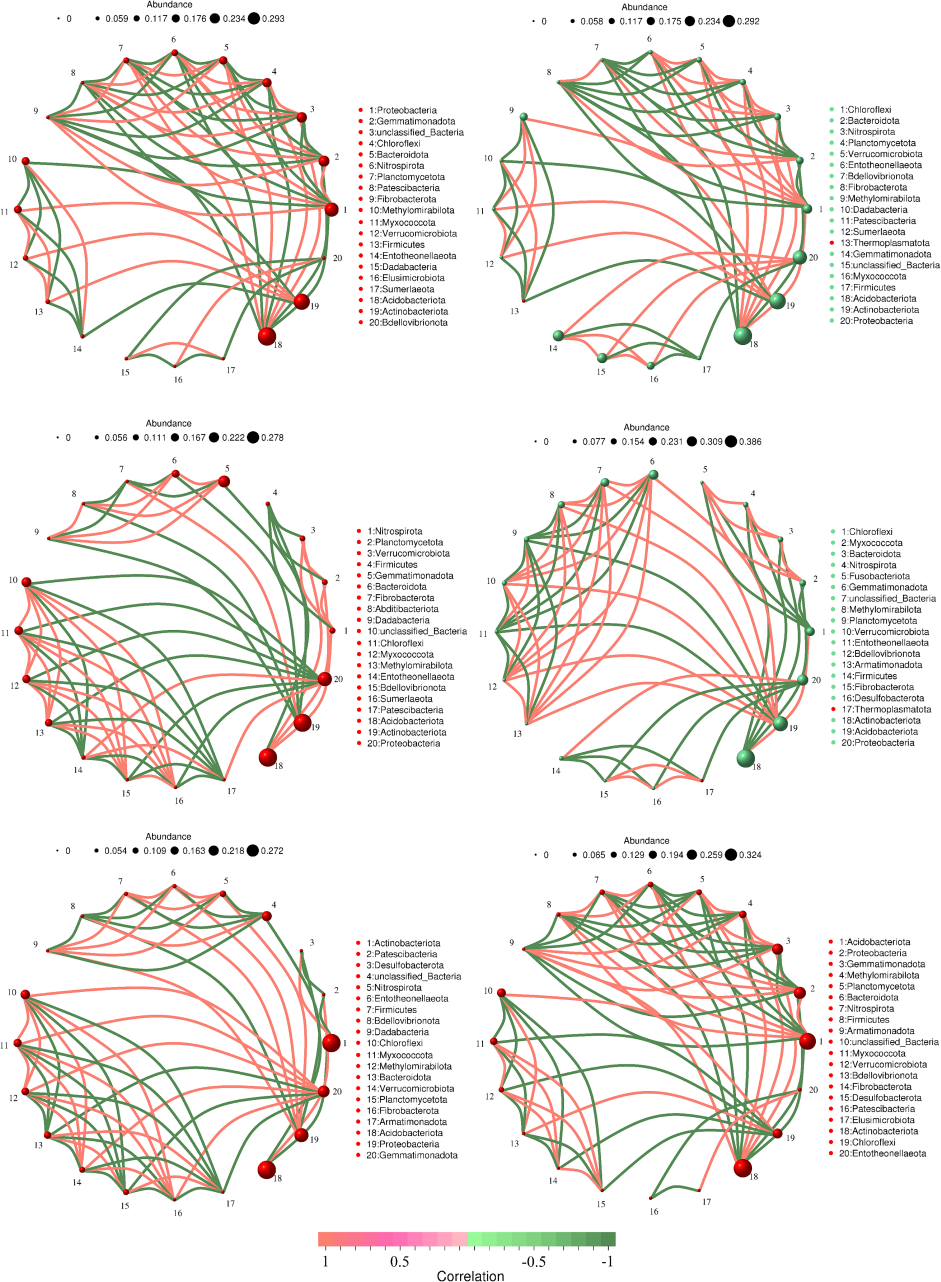
The soil bacterial network in the core protected zone **(A)**, general controlled zone **(C)** and outside the protected areas **(E)** in shrub grassland and the soil bacterial network in the core protected zone **(B)**, general controlled zone **(D)** and outside the protected areas **(F)** in herbaceous grassland.

To investigate the differences in soil bacterial community structure inside and outside the nature reserve, we compared the correlation networks of the top 20 bacterial phyla in relative abundance. In shrub grassland, the core phylum of the soil bacterial network in the core protected zones, general controlled zones and outside the protected areas were Acidobacteriota, Proteobacteria and Gemmatimonadota, respectively (Figures 5A,C,E). There were 29, 31, and 31 sets of positive correlations and 37, 30, and 32 sets of negative correlations between soil bacterial phyla in core protected areas, general control areas, and outside protected areas, respectively. There was a significant positive correlation between soil Acidobacteriota and Nitrospirota in core protected areas and general control areas within nature reserves (Figures 5A,C), while there was no significant correlation between the two in soils outside protected areas (Figure 5E).

In herbaceous grassland, the core phyla of the soil bacterial network in the core protected zones, general controlled zones and outside the protected areas were Acidobacteriota, Acidobacteriota and Actinobacteriota, respectively (Figures 5B,D,F). There were 31, 33, and 30 sets of positive correlations and 30, 28, and 39 sets of negative correlations between soil bacterial phyla in the core protected area, general control area, and outside the protected area, respectively. There was a significant negative correlation between soil Actinobacteriota and Acidobacteriota outside the protected area (Figures 5B,D), but there was no significant correlation between the two in the soil of the core protected area and the general control area within the nature reserve (Figure 5F).

### 3.3 Correlation between soil bacterial communities and plant biomass and soil chemical indices

**Figure 6:**
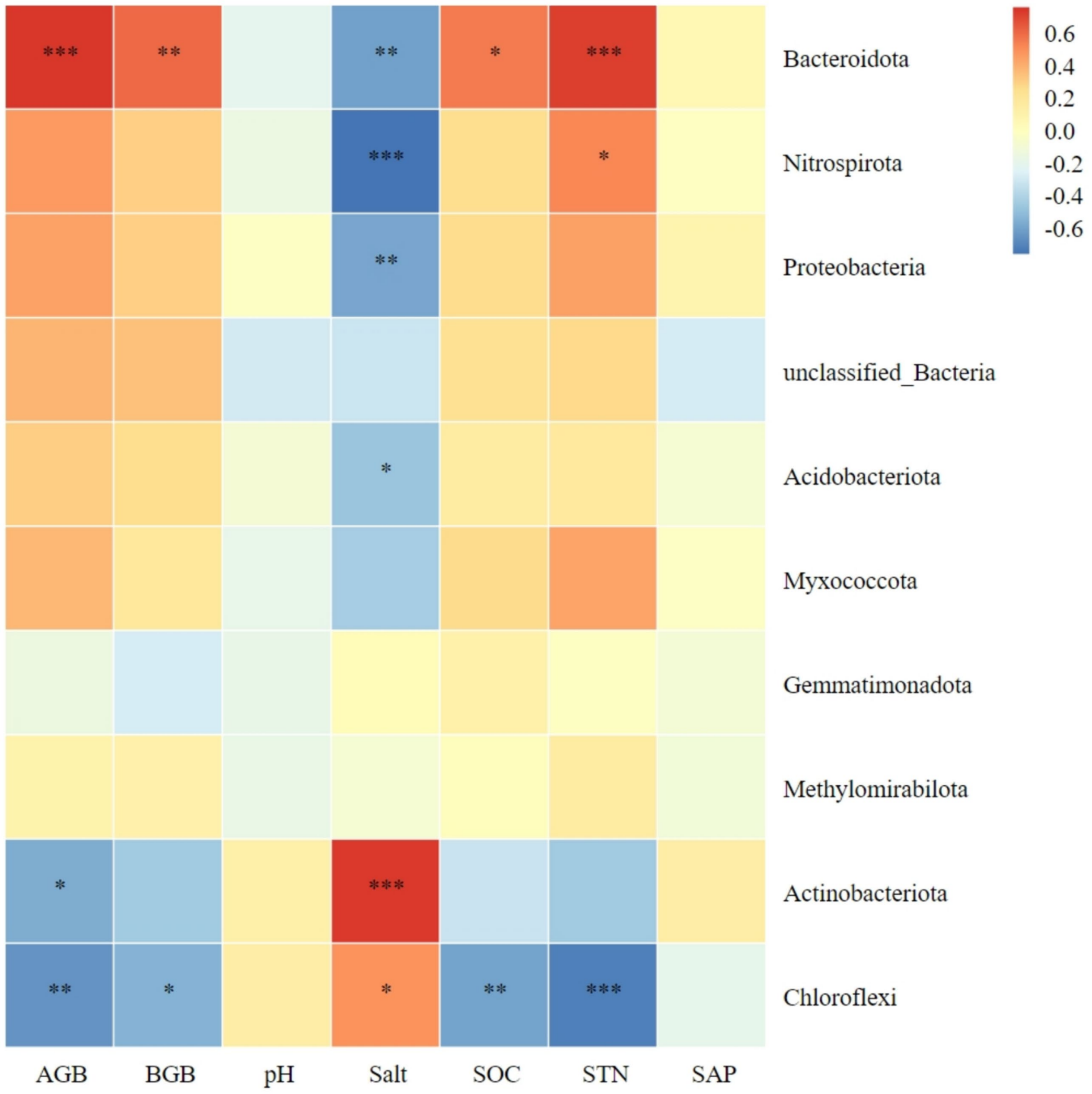
Correlation heatmap of soil bacterial phyla with plant biomass and soil chemical index. AGB, aboveground biomass; BGB, belowground biomass; SOC, soil organic carbon; STN, soil total nitrogen; SAP, soil active phosphorus. *Significance level *P* < 0.05, **Significance level *P* < 0.01, ***Significance level *P* < 0.001.

Figure 6 shows the relationship between the top 10 bacterial phyla in abundance and plant biomass, as well as soil factors. There was a significant positive correlation between Bacteroidota in soil and both aboveground and belowground plant biomass. In contrast, Chloroflexi showed a negative correlation with both plants aboveground and belowground biomass. Actinobacteriota was negatively correlated only with aboveground plant biomass. The aboveground and belowground biomass of plants showed a negative correlation with Chloroflexi. Meanwhile, Actinobacteriota and Chloroflexi exhibited a positive correlation with soil salt content. On the other hand, Bacteroidota, Nitrospirota, Proteobacteria, and Acidobacteriota showed a negative correlation with soil salt content. Additionally, there was a significant positive correlation between Bacteroidota and soil organic carbon. Lastly, Chloroflexi exhibited a negative correlation with soil organic carbon. The abundance of Bacteroidota and Nitrospirota showed a positive correlation with soil total nitrogen, while Chloroflexi exhibited a negative correlation. No significant correlation was observed between the dominant bacteria and soil pH or available phosphorus (Figure 6).

**Figure 7:**
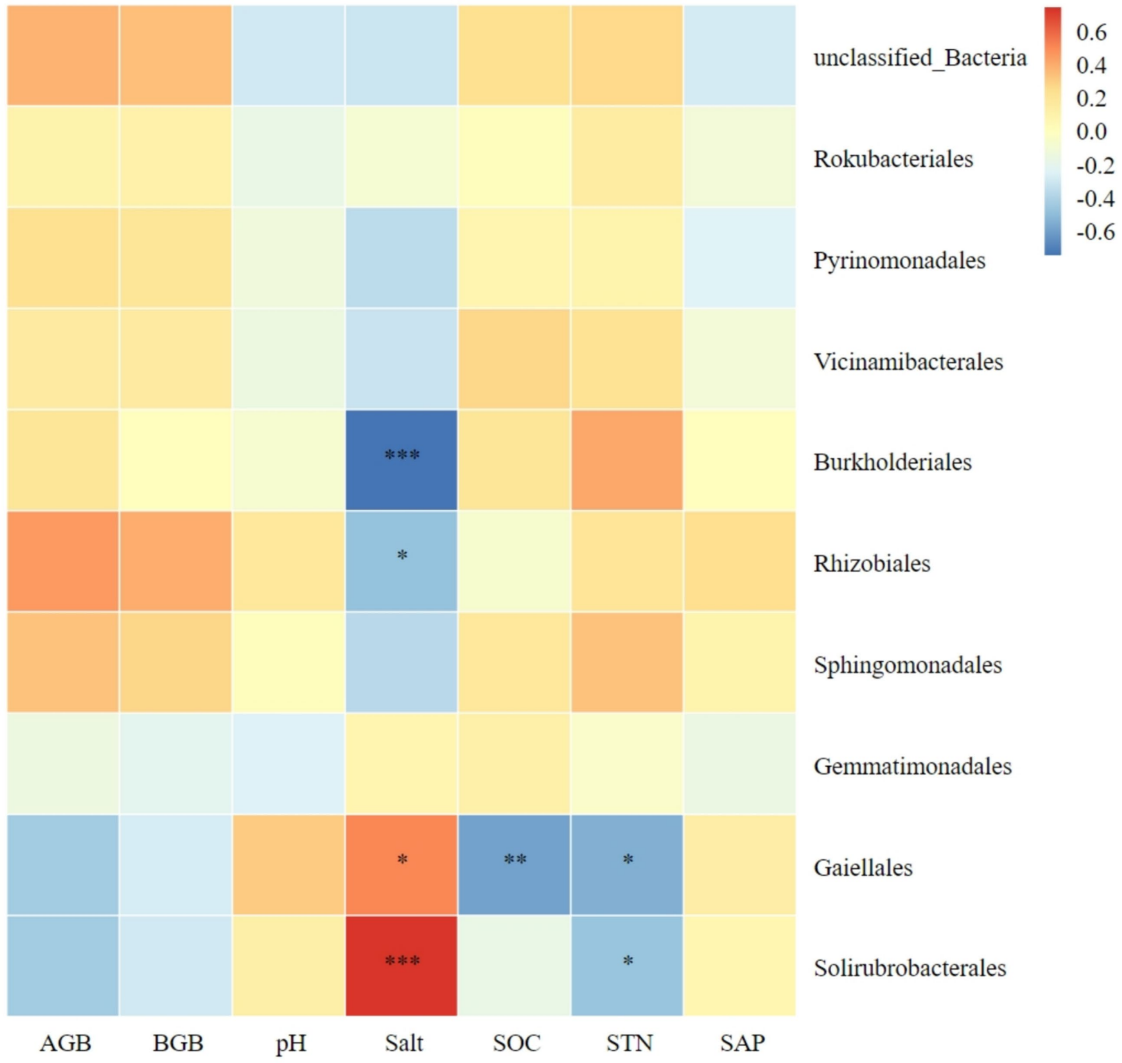
Correlation heatmap of soil bacterial orders with plant biomass and soil chemical index. AGB, aboveground biomass; BGB, belowground biomass; SOC, soil organic carbon; STN, soil total nitrogen; SAP, soil active phosphorus. *Significance level *P* < 0.05, **Significance level *P* < 0.01, ***Significance level *P* < 0.001.

The relationship between the 10 most abundant bacterial orders and plant biomass, as well as soil factors, is illustrated in Figure 7. Both Solirubrobacterales and Gaiellales in soil were significantly positively correlated with soil salt content; while both Rhizobiales and Burkholderiales were significantly negatively correlated with soil salt content. Gaiellales was significantly negatively correlated with soil organic carbon. Both Solirubrobacterales and Gaiellales were significantly negatively correlated with soil total nitrogen.

## 4 Discussion

### 4.1 Differences in dominant bacterial phyla, genus and α-diversity of soil bacterial communities in and outside nature reserves

In this study, the soil dominant bacterial phyla in the nature reserve were more similar to that outside the nature reserve, which were Acidobacteriota, Actinobacteriota, Bacteroidota, Chloroflexi, Gemmatimonadota, Methylomirabilota, Myxococcota, Nitrospirota, Proteobacteria, and unclassified_Bacteria; the relative abundance of each dominant phylum varied slightly. In herbaceous grassland, the highest abundance of soil Acidobacteriota was found in the core protected zone within the protected zone (29.21% ± 2.42%), while the highest abundance of soil Actinobacteriota was found in the general controlled zones and the soil outside the protected areas (38.58% ± 3.84 and 32.36% ± 4.76%, respectively), which suggests that the strict protective measures altered relative abundance of soil dominant phyla in herbaceous grassland. Acidobacteriota plays an important role in degrading plant residues and participating in ecosystem iron cycling (Eichorst et al., 2011; Stursová et al., 2012). The increase in the relative abundance of soil Acidobacteriota under strict protection measures favors the decomposition and transformation of organic matter in plant communities and promotes soil nutrient cycling. Species diversity is an indispensable factor in maintaining ecosystem function, and for soil ecosystems, soil bacterial community diversity plays an irreplaceable role in promoting soil material cycling and energy flow (Lladó et al., 2017; Yu et al., 2021). The Chao1 index is a crucial metric for characterizing the species richness of soil bacterial communities (Wang et al., 2023). Our study revealed that in shrub grasslands, the Chao1 index of soil bacterial communities in core protected zones and general controlled zones was significantly higher than that outside the protected areas (*p* < 0.05). This increase can be attributed to the protective measures implemented by nature reserves. Human activities, such as grazing, were effectively restricted in the protected areas of desert grassland. This restriction led to the effective restoration of vegetation and soil (Huang et al., 2021). As a result, soil bacterial communities were able to thrive, leading to an increase in species richness. The Shannon index of soil bacterial communities in the herbaceous grassland was higher in the core protected zone than outside of it (*p* < 0.05). This suggests that management measures in protected zones have a positive effect on improving the diversity of soil bacterial communities. However, the changes in the patterns of the Chao1 index and Shannon index of soil bacterial communities inside and outside the protected areas under different vegetation types (shrub grassland and herbaceous grassland) were not entirely consistent. This suggests that there are differences in the species richness and diversity of soil bacterial communities among different vegetation types under the same management measures. These differences may be related to the protective effect of scrub, the “fertility islands effect,” and micro environmental improvement (Gregory et al., 2001; Chen et al., 2015). Further investigation is needed to understand the mechanisms behind these differences.

### 4.2 Comparison of differences in soil bacterial community structure inside and outside the nature reserves

The analysis of principal components reveals that the soil bacterial community structure in the shrub grassland within the nature reserve did not differ significantly from that outside the reserve. The distance between each sample point was relatively far, which may be attributed to the spatial heterogeneity of the shrub grassland (Cheng et al., 2024). In herbaceous grassland, the soil bacterial community structure inside the protected area differed significantly from that outside, indicating that the management measures implemented in the protected area had a significant impact on the soil bacterial community structure of herbaceous grassland. This finding is consistent with the results of the study conducted by Esposito (Esposito et al., 2024). In desert grasslands, the primary human activity is grazing on herbaceous grasses. Overgrazing can damage both the vegetation and soil structure and function (Milazzo et al., 2023), which can then impact the structure of the soil bacterial community. Taxa with high connectivity are typically considered core taxa in the network and play a crucial role in the bacterial network (Ma B. et al., 2018). The correlation network analysis among soil dominant bacterial phyla in this study reveals that in shrub grassland, the core phyla of soil bacterial networks in core protected zones, general controlled zones, and outside protected zones were Acidobacteriota, Proteobacteria, and Gemmatimonadota, respectively. In herbaceous grassland, the soil bacterial networks in core protected zones, general controlled zones, and outside protected zones of nature reserves were Acidobacteria, Proteobacteria, and Gemmatimonadota, respectively. In herbaceous grasslands, the Acidobacteriota phylum dominated the core protected zones, while the general control zones were dominated by Acidobacteriota as well. Actinobacteriota was the dominant phylum in the outside protected zones of nature reserves. The core phyla of the soil bacterial network structure changed as the protection measures became stricter (Qiu et al., 2022). The core phylum of the soil bacterial network structure of different vegetation types in the core protected zone was Acidobacteriota, while the core phylum of the soil bacterial network structure of different vegetation types in the general controlled zone and outside the protected areas was different, which may be due to the different intensities of human activities in the two zones (Hua et al., 2024). The correlation between various types of soil bacteria also changes with the environment, and it has been found that the correlation between various types of soil bacteria in different stages of soil development showed significant differences due to different environments (Dini-Andreote et al., 2014). In this study, there were 29, 31, and 31 sets of positive correlations and 37, 30, and 32 sets of negative correlations between soil bacterial phyla in core protected areas, general control areas, and outside protected areas, respectively. However, in herbaceous grassland, the correlations between soil bacterial phyla were different of that in shrub grassland, which was related to the habitat differences between shrub and herbaceous grassland. We need to collect more samples to improve further research. And we need to conduct in-depth analysis of correlation networks in the future, including conducting research at the OTU level.

### 4.3 Correlation between soil bacterial communities and plant biomass and soil chemical indices

Research has demonstrated that an increase in litter amount of plant community and belowground biomass within plant communities can lead to an increase in soil nutrient content and promote soil microbial diversity (Mao et al., 2024). Microorganisms require nutrients mainly derived from soil. Studies have shown that when soil nutrients are abundant, the number and biomass of microbial communities are positively correlated with soil organic carbon and nitrogen, as well as total soil nitrogen (He et al., 2023). Additionally, these studies have found a correlation between soil bacterial communities and plant productivity. Our study revealed that Bacteroidota, Nitrospirota, Proteobacteria, and Acidobacteriota are significantly negatively correlated with soil salinity. This suggests that high salinity soil environments have a negative effect on these bacterial phyla, which is unfavorable to their growth and development, and negatively affects the structure of the entire bacterial community. Bacteroidota exhibited significant positive correlations with aboveground biomass, belowground biomass, soil organic carbon, and soil total nitrogen. The vegetation and soil restoration measures were found to be favorable for the survival and development of Bacteroidota. In this study, Chloroflexi exhibited significant negative correlations with plant aboveground biomass, belowground biomass, and soil organic carbon. This finding contrasts with the results of (Zhang et al., 2024) in Jilin Momog, which may be due to differences in the response of soil Chloroflexi to plant productivity and soil factors in arid versus humid environments. We found that higher plant productivity and soil nutrients had a positive effect on most soil dominant phyla, while higher soil pH and salinity had a negative effect on most soil dominant phyla.

## 5 Conclusion

The species richness and diversity of soil bacterial communities are higher inside than outside the West Ordos National Nature Reserve. Higher plant productivity and soil nutrients have positive effects on most soil dominant bacterial phyla, while higher soil pH and salinity have negative effects on most soil dominant bacterial phyla. Therefore, improving the management of nature reserves and implementing measures to restore saline and alkaline lands in protected areas can benefit the survival and development of soil bacterial communities, promoting the sustainable development of soil ecosystems.

## Data availability statement

The original contributions presented in the study are included in the article/supplementary material, further inquiries can be directed to the corresponding authors.

## Author contributions

PG: Conceptualization, Investigation, Software, Writing – original draft, Writing – review & editing. SL: Investigation, Software, Writing – original draft. JZ: Methodology, Writing – review & editing. QL: Methodology, Supervision, Writing – review & editing.

## References

[ref1] Agbenin J. O. Adeniyi T. (2014). The microbial biomass properties of a savanna soil under improved grass and legume pastures in northern Nigeria. Agric. Ecosyst. Environ. 193, 9–24. doi: 10.1016/j.agee.2005.03.003

[ref2] Atwell M. A. Wuddivira M. N. Wilson M. (2018). Sustainable management of tropical small island ecosystems for the optimization of soil natural capital and ecosystem services: a case of a Caribbean soil ecosystem-Aripo savannas Trinidad. J. Soils Sediments 18, 1654–1667. doi: 10.1007/s11368-017-1865-3

[ref3] Caporaso J. G. Bittinger K. Bushman F. D. DeSantis T. Z. Andersen G. L. Knight R. (2010). PyNAST: a flexible tool for aligning sequences to a template alignment. Bioinformatics 26, 266–267. doi: 10.1093/bioinformatics/btp636, PMID: 19914921 PMC2804299

[ref4] Caporaso J. G. Kuczynski J. Stombaugh J. Bittinger K. Bushman F. D. Costello E. K.. (2013). QIIME allows analysis of high-throughput community sequencing data. Nat. Methods 10:996. doi: 10.1038/nmeth.f.303 20383131 PMC3156573

[ref5] Carranza T. Balmford A. Kapos V. Manica A. (2013). Protected area effectiveness in reducing conversion in a rapidly vanishing ecosystem: the Brazilian Cerrado. Conserv. Lett. 7, 216–223. doi: 10.1111/conl.12049

[ref6] Chai Y. Cao Y. Yue M. Tian T. Yin Q. Dang H.. (2019). Soil abiotic properties and plant functional traits mediate associations between soil microbial and plant communities during a secondary forest succession on the Loess Plateau. Front. Microbiol. 10:895. doi: 10.3389/fmicb.2019.00895 31105679 PMC6499021

[ref7] Chen L. Li H. Zhang P. Zhao X. Zhou L. Liu T.. (2015). Climate and native grassland vegetation as drivers of the community structures of shrub-encroached grasslands in Inner Mongolia, China. Landscape Ecol. 30, 1627–1641. doi: 10.1007/s10980-014-0044-9

[ref8] Cheng L. Wu B. Pang Y. Jia X. (2024). Shrub growth improves morphological features of Nebkhas: a case study of *Nitraria tangutorum* in the Tengger Desert. Plants 13:624. doi: 10.3390/plants13050624, PMID: 38475471 PMC10934661

[ref9] Crepin J. Johnson L. R. (1993). Soil sampling and methods of analysis. J. Environ. Qual. 38, 15–24.

[ref10] Dini-Andreote F. Silva M. D. P. E. Triadó-Margarit X. Casamayor E. O. van Elsas J. D. Salles J. F. (2014). Dynamics of bacterial community succession in a salt marsh chronosequence: evidences for temporal niche partitioning. ISME J. 8, 1989–2001. doi: 10.1038/ismej.2014.54 24739625 PMC4184019

[ref11] Dong X. Xu D. Wang D. Han C. Huang Y. Zhang J. (2023). Leaf–root–soil stoichiometric characteristics in different shrub ages of *Ammopiptanthus mongolicus*. Plants 12:3103. doi: 10.3390/plants12173103, PMID: 37687349 PMC10490301

[ref12] Eichorst S. A. Kuske C. R. Schmidt T. M. (2011). Influence of plant polymers on the distribution and cultivation of bacteria in the phylum Acidobacteria. Appl. Environ. Microb. 77, 586–596. doi: 10.1128/AEM.01080-10, PMID: 21097594 PMC3020536

[ref13] Esposito A. Del Duca S. Vitali F. Bigiotti G. Mocali S. Semenzato G.. (2024). The great Gobi A strictly protected area: characterization of soil bacterial communities from four oases. Microorganisms 12:320. doi: 10.3390/microorganisms12020320, PMID: 38399724 PMC10891509

[ref14] Gray C. L. Hill S. L. L. Newbold T. Hudson L. N. Börger L. Contu S.. (2016). Local biodiversity is higher inside than outside terrestrial protected areas worldwide. Nat. Commun. 7:12306. doi: 10.1038/ncomms12306, PMID: 27465407 PMC4974472

[ref15] Gregory S. O. Bruce M. William H. S. (2001). Degradation of sandy arid shrubland environments: observations, process modelling, and management implications. J. Arid Environ. 47, 123–144. doi: 10.1006/jare.2000.0711

[ref16] Griffiths B. S. Hallett P. D. Kuan H. L. Gregory A. S. Watts C. W. Whitmore A. P. (2008). Functional resilience of soil microbial communities depends on both soil structure and microbial community composition. Biol. Fertil. Soils 44, 745–754. doi: 10.1007/s00374-007-0257-z

[ref17] Guo X. Zuo X. Yue P. Li X. Hu Y. Chen M.. (2022). Direct and indirect effects of precipitation change and nutrients addition on desert steppe productivity in Inner Mongolia, northern China. Plant Soil 471, 527–540. doi: 10.1007/s11104-021-05206-2

[ref18] He L. Sun X. Li S. Zhou W. Chen Z. Bai X. (2023). The vertical distribution and control factor of microbial biomass and bacterial community at macroecological scales. Sci. Total Environ. 869:161754. doi: 10.1016/j.scitotenv.2023.161754, PMID: 36709888

[ref19] Hua H. Sui X. Liu Y. Liu X. Chang Q. Xu R.. (2024). Effects of land use type transformation on the structure and diversity of soil bacterial communities. Life 14:252. doi: 10.3390/life14020252, PMID: 38398761 PMC10890093

[ref20] Huang H. Zhang K. Zhang B. Liu S. Chu H. Qi Y.. (2021). Analysis on the relationship between winter precipitation and the annual variation of horse stomach fly community in arid desert steppe, Northwest China (2007–2019). Integr. Zool. 17, 128–138. doi: 10.1111/1749-4877.12578 34254452 PMC9291967

[ref21] Jones W. J. (2010). High-throughput sequencing and metagenomics. Estuar. Coast 33, 944–952. doi: 10.1007/s12237-009-9182-8

[ref22] Li E. G. Huang Y. M. Chen H. Y. Zhang J. H. (2018). Floristic diversity analysis of the Ordos Plateau, a biodiversity hotspot in arid and semi-arid areas of China. Folia Geobot. 53, 405–416. doi: 10.1007/s12224-018-9331-6

[ref23] Li G. Kim S. Han S. Chang H. Du D. Son Y. (2018). Precipitation affects soil microbial and extracellular enzymatic responses to warming. Soil Biol. Biochem. 120, 212–221. doi: 10.1016/j.soilbio.2018.02.014

[ref24] Liu N. Kan H. Yang G. Zhang Y. (2016). Changes in plant, soil, and microbes in a typical steppe from simulated grazing: explaining potential change in soil C. Ecol. Monogr. 85, 269–286. doi: 10.1890/14-1368.1

[ref25] Liu J. Sui Y. Yu Z. Shi Y. Chu H. Jin J.. (2014). High throughput sequencing analysis of biogeographical distribution of bacterial communities in the black soils of Northeast China. Soil Biol. Biochem. 70, 113–122. doi: 10.1016/j.soilbio.2013.12.014

[ref26] Lladó S. López-Mondéjar R. Baldrian P. (2017). Forest soil bacteria: diversity, involvement in ecosystem processes, and response to global change. Microbiol. Mol. Biol. Rev. 81:e00063. doi: 10.1128/MMBR.00063-16 28404790 PMC5485800

[ref27] Ma L. Pang D. Gao J. Wang W. Sun R. (2023). Ecological asset assessment and ecological compensation standards for desert nature reserves: evidence from three different climate zones in China. Sustainability 15:10679. doi: 10.3390/su151310679

[ref28] Ma S. Verheyen K. Props R. Wasof S. Vanhellemont M. Boeckx P.. (2018). Plant and soil microbe responses to light, warming and nitrogen addition in a temperate forest. Funct. Ecol. 32, 1293–1303. doi: 10.1111/1365-2435.13061

[ref29] Ma B. Zhao K. Lv X. Su W. Dai Z. Gilbert J. A.. (2018). Genetic correlation network prediction of forest soil microbial functional organization. ISME J. 12, 2492–2505. doi: 10.1038/s41396-018-0232-8, PMID: 30046166 PMC6155114

[ref30] Magoc T. Salzberg S. L. (2011). FLASH: fast length adjustment of short reads to improve genome assemblies. Bioinformatics 27, 2957–2963. doi: 10.1093/bioinformatics/btr507, PMID: 21903629 PMC3198573

[ref31] Mao Z. Yue M. Wang Y. Li L. Li Y. (2024). Soil microorganisms mediated the responses of the plant–soil systems of *Neotrinia splendens* to nitrogen addition and warming in a desert ecosystem. Agronomy 14:132. doi: 10.3390/agronomy14010132

[ref32] Milazzo F. Francksen R. M. Abdalla M. Ravetto Enri S. Zavattaro L. Pittarello M.. (2023). An overview of permanent grassland grazing management practices and the impacts on principal soil quality indicators. Agronomy 13:1366. doi: 10.3390/agronomy13051366

[ref33] Mlambo D. Mwenje E. Nyathi P. (2007). Effects of tree cover and season on soil nitrogen dynamics and microbial biomass in an African savanna woodland dominated by *Colophospermum mopane*. J. Trop. Ecol. 23, 437–448. doi: 10.1017/S0266467407004233

[ref34] Otasek D. Morris J. H. Bouças J. Pico A. R. Demchak B. (2019). Cytoscape automation: empowering workflow-based network analysis. Genome Biol. 20:185. doi: 10.1186/s13059-019-1758-4, PMID: 31477170 PMC6717989

[ref35] Qiu Z. Shi C. Zhang M. Tang L. Li X. Zhao T.. (2022). Changes in soil bacterial community structure and diversity of *Pinus tabuliformis* plantation after 65 years of near-naturalization in North China. J Sustain. For. 42, 887–909. doi: 10.1080/10549811.2022.2123359

[ref36] Sankey J. Ravi S. Wallace C. S. A. Webb R. H. Huxman T. E. (2015). Quantifying soil surface change in degraded drylands: shrub encroachment and effects of fire and vegetation removal in a desert grassland. J. Geophys. Res. Biogeosci. 117, 194–215. doi: 10.1029/2012JG002002

[ref37] Stursová M. Zifcáková L. Leigh M. B. Burgess R. Baldrian P. (2012). Cellulose utilization in forest litter and soil: identification of bacterial and fungal decomposers. FEMS Microbiol. Ecol. 80, 735–746. doi: 10.1111/j.1574-6941.2012.01343.x, PMID: 22379979

[ref38] Sünnemann M. Siebert J. Reitz T. Schädler M. Yin R. Eisenhauer N. (2021). Combined effects of land-use type and climate change on soil microbial activity and invertebrate decomposer activity. Agric. Ecosyst. Environ. 318:107490. doi: 10.1016/j.agee.2021.107490

[ref39] Teng Z. D. Zhu Y. D. Li M. Whelan M. J. (2018). Microbial community composition and activity controls phosphorus transformation in rhizosphere soils of the Yeyahu wetland in Beijing, China. Sci. Total Environ. 628, 1266–1277. doi: 10.1016/j.scitotenv.2018.02.115 30045548

[ref40] Tranquilli S. Abedi-Lartey M. Amsini F. Arranz L. Asamoah A. Babafemi O.. (2012). Lack of conservation effort rapidly increases African great ape extinction risk. Conserv. Lett. 5, 48–55. doi: 10.1111/j.1755-263X.2011.00211.x

[ref41] Wang M. Yu X. Weng X. Zeng X. Li M. Sui X. (2023). Meta-analysis of the effects of biochar application on the diversity of soil bacteria and fungi. Microorganisms 11:641. doi: 10.3390/microorganisms11030641, PMID: 36985214 PMC10057247

[ref42] Wei Y. Liu M. Xie Y. Gao Y. Liu M. Yue P.. (2024). Study on soil organic carbon of plant communities in enclosed area of Xilamuren desert grassland. Res. Soil Water Conserv. 31, 35–43. doi: 10.13869/j.cnki.rswc.2024.01.015

[ref43] Xia Q. Rufty T. Shi W. (2020). Soil microbial diversity and composition: links to soil texture and associated properties. Soil Biol. Biochem. 149:107953. doi: 10.1016/j.soilbio.2020.107953

[ref44] Xiong D. Shi P. Zhang X. Zou C. (2016). Effects of grazing exclusion on carbon sequestration and plant diversity in grasslands of China a meta-analysis. Ecol. Eng. 94, 647–655. doi: 10.1016/j.ecoleng.2016.06.124

[ref45] Yang Z. Zhu Q. Zhan W. Xu Y. Zhu E. Gao Y.. (2018). The linkage between vegetation and soil nutrients and their variation under different grazing intensities in an alpine meadow on the eastern Qinghai-Tibetan plateau. Ecol. Eng. 110, 128–136. doi: 10.1016/j.ecoleng.2017.11.001

[ref46] Yu Y. Liu L. Wang J. Zhang Y. Xiao C. (2021). Effects of warming on the bacterial community and its function in a temperate steppe. Sci. Total Environ. 792:148409. doi: 10.1016/j.scitotenv.2021.148409, PMID: 34146803

[ref47] Yu C. Y. Zhang Z. H. Jeppesen E. Gao Y. Liu Y. X. Liu Y. J.. (2024). Assessment of the effectiveness of China’s protected areas in enhancing ecosystem services. Ecosyst. Serv. 65:101588. doi: 10.1016/j.ecoser.2023.101588

[ref48] Zhang Y. Gao X. Yuan Y. Hou L. Dang Z. Ma L. (2023). Plant and soil microbial diversity co-regulate ecosystem multifunctionality during desertification in a temperate grassland. Plants 12:3743. doi: 10.3390/plants12213743, PMID: 37960099 PMC10649343

[ref49] Zhang Y. Miao S. Song Y. Wang X. Jin F. (2024). Biochar application reduces saline–alkali stress by improving soil functions and regulating the diversity and abundance of soil bacterial community in highly saline–alkali paddy field. Sustainability 16:1001. doi: 10.3390/su16031001

[ref50] Zuo X. Li X. Yue P. Guo A. Yue X. Xu C.. (2022). Drought-driven shifts in relationships between plant biodiversity and productivity in temperate steppes. Funct. Ecol. 36, 2917–2928. doi: 10.1111/1365-2435.14219

